# Health professionals’ and beauty therapists’ perspectives on female genital cosmetic surgery: an interview study

**DOI:** 10.1186/s12905-023-02744-y

**Published:** 2023-11-14

**Authors:** Maggie Kirkman, Amy Dobson, Karalyn McDonald, Amy Webster, Pramasari Wijaya, Jane Fisher

**Affiliations:** 1 Global and Women’s Health, Monash University, Public Health and Preventive Medicine, 553 St Kilda Road, Melbourne, 3004 Australia; 2 Institute for Advanced Studies in the Humanities, University of Queensland, Brisbane, Australia; 3 Women’s Health Victoria, Melbourne, Australia

**Keywords:** Female Genital Cosmetic Surgery, Qualitative research, Interviews, Medical practitioners, Medical Education, Australia

## Abstract

**Background:**

Female genital cosmetic surgery (FGCS) changes the structure and appearance of healthy external genitalia. We aimed to identify discourses that help explain and rationalise FGCS and to derive from them possibilities for informing clinical education.

**Methods:**

We interviewed 16 health professionals and 5 non-health professionals who deal with women’s bodies using a study-specific semi-structured interview guide. We analysed transcripts using a three-step iterative process: identifying themes relevant to indications for FGCS, identifying the discourses within which they were positioned, and categorising and theorising discourses.

**Results:**

We identified discourses that we categorised within four themes: *Diversity and the Normal Vulva* (diversity was both acknowledged and rejected); *Indications for FGCS* (Functional, Psychological, Appearance); *Ethical Perspectives*; and *Reasons Women Seek FGCS* (Pubic Depilation, Media Representation, Pornography, Advertising Regulations, Social Pressure, Genital Unfamiliarity).

**Conclusions:**

Vulvar aesthetics constitute a social construct to which medical practice and opinion contribute and by which they are influenced; education and reform need to occur on all fronts. Resources that not only establish genital diversity but also challenge limited vulvar aesthetics could be developed in consultation with women, healthcare practitioners, mental health specialists, and others with knowledge of social constructs of women’s bodies.

Female genital cosmetic surgery (FGCS) is a term encompassing non-medically indicated surgical procedures that change the structure and appearance of women’s healthy external genitalia [1–3]. Procedures include reduction of the labia minora (labiaplasty/labioplasty, the most common procedure) and clitoral hood, ‘plumping’ of the labia majora, and liposuction of the mons pubis.

Professional medical bodies in the United States [1], United Kingdom [3], Canada [4], and Australia [5] have expressed concern about adverse outcomes and inadequate clinical guidelines associated with FGCS. All emphasise the lack of evidence for claims of improvement in self-image or sexual function and note potential adverse outcomes including scarring, permanent disfigurement, infection, altered sexual sensation, and painful sexual intercourse. ACOG [1] also calls for obstetrician-gynaecologists to be equipped to discuss normal sexual development, the wide variability in appearance of genitalia, non-surgical treatment options, and autonomous decision making.

Despite the lack of comprehensive data, it is evident that FGCS has increased in popularity over the last two decades [1]. In Australia, records are kept for procedures paid for under the national healthcare scheme, Medicare. A report by the Australian Government Department of Health [6] recorded an increase in the number of claims for Medicare Benefits Schedule item 35,533 (vulvoplasty and labiaplasty), especially in New South Wales (31%) and Victoria (25%). However, since 2014, procedures without medical indication are not publicly funded and no recent data are therefore available [7].

The idealised aesthetic standard for women’s genitals, at least in the West, has been identified as a hairless, symmetrical “clean slit”, without protruding labia minora or clitoris [8], also labelled the Barbie Doll ideal [9]. It is consistent with this assessment that women have been found to use the words “smooth” and “not sticking out” to describe “normal” genital appearance [10]. Although their genitals are within the normal range of labia minora width and length, women may still seek FGCS to conform to the ideal [11] while acknowledging social pressure to do so [12]. It has been argued that the internet, social media, and a consumerist culture have contributed to the demand for the ideal, minimalist vulva [13], along with cultural trends in pubic hair removal, the desire to differentiate female from male genitals, and limited vulvar diversity in published images [14]. Women in the US and Australia report using their physicians to learn about vulvar appearance, along with the internet, pornography, and school sex education classes [10, 15]. Medical practitioners therefore play an important role in women’s genital knowledge and satisfaction.

Advertisements for FGCS in Australia [16, 17] and elsewhere [18, 19] reveal the perspectives of some medical practitioners. Despite warnings by professional organisations, websites advertising FGCS depict the procedure as simple and safe with minimal risks [16, 18]. They tend to pathologise the healthy vulva by using terms such as ‘labial hypertrophy’, implying that visible labia minora or clitoris render the vulva not only uncomfortable but too masculine [16]. The point at which labia become ‘hypertrophic’ is rarely noted, although 75% of surveyed sexual medicine specialists were found to state that 50 mm was the maximum normal length [20]. Variation of the labia minora has been recorded at lengths of 5-100 mm and of the clitoris at 0.5–34 mm [1]; ACOG specifies that measures outside these ranges do not indicate abnormality. Some medical websites acknowledge diverse genital appearance but nevertheless recommend surgery to conform to a feminine genital aesthetic, for a ‘youthful’ appearance, to improve (hetero)sexual function, and to boost self-esteem [16, 18, 19]. It is also claimed, without evidence, that ‘large’ labia minora cause recurrent infections and an unpleasant odour that will be cured by labiaplasty [16, 18]. Advertisements extend to revising ‘botched’ labiaplasty, implying that satisfaction is possible (with a better surgeon) without identifying problems inherent in the procedure [21].

Healthcare providers’ opinions on female genital aesthetics and FGCS have been found to vary by gender and specialty, with men more prepared to perform surgery than women and plastic surgeons having a more limited aesthetic than GPs and gynaecologists [22]. Adolescent specialists were more likely to offer reassurance about large labia minora whereas gynaecologists who did not specialise in adolescents were more likely to offer labiaplasty [23]. The reasons given by physicians for FGCS are consistent with those identified in websites advertising the procedures: to reduce discomfort, increase self-esteem, and improve sexual satisfaction [19, 20, 23, 24]. Clinicians evaluating their own patients tend to find high satisfaction in improved body image and sexual function [24]. GPs in Australia were found to believe that psychological problems lay behind requests for FGCS and tended to say that they needed more information to enable them to support their patients in this matter [25, 26].

As part of a multimethod study designed to elucidate sociocultural aspects of the increasing demand for FGCS by women and girls in Australia, we consulted healthcare practitioners and other professionals whose work involves women’s bodies about their experience and opinions of genital aesthetics and FGCS. Our aim was to identify discourses that explained or justified female genital cosmetic surgery and to derive from them possibilities for informing clinical education.

## Method

Eligible participants were health professionals (gynaecologists, plastic surgeons, cosmetic surgeons, GPs, nurses, medical students, pelvic floor physiotherapists) and non-health professionals who deal with women’s bodies (beauty therapists, body piercers) who could communicate in English. We called for volunteers with notices on the websites and in newsletters of the potential volunteers’ professional organisations, followed by purposive recruitment to increase gender and disciplinary diversity; this included placing notices in beauty salons and distributing flyers to body piercers, physiotherapists, and clinics staffed by men. Our goal was to find as broad a range of perspectives as we could. We offered a choice of semi-structured interviews by telephone, in person, or by email, and sent an Explanatory Statement and Consent Form to those who expressed interest in participating. Participants could choose to give consent by signing and returning the form or orally (audio recorded) at the beginning of the interview.

A study-specific interview guide was developed, covering the topics of why women and girls might be seeking genital cosmetic surgery, how participants do or would respond to questions from women and girls about whether their genitals are normal and the need for cosmetic surgery, experience with female genital cosmetic surgery, and reflections on what colleagues say about genital cosmetic surgery. Questions were adapted by the interviewer to each participant’s profession and experience. The interview was conversational and encouraged participants to expand on matters important to them. The interviewer (KM or MK) defined female genital cosmetic surgery as surgically changing the appearance of a women’s genitals for cosmetic reasons, not because of a medical condition. We thus excluded from consideration vaginal tightening and injury repair.

Interviews were audio-recorded and transcribed. Transcripts and email text were edited to obscure identifying details; identification codes were allocated to each participant. We analysed using a three-step iterative process: identifying themes relevant to indications for FGCS, identifying the discourses within which they were positioned, and categorising and theorising discourses [27, 28].

## Results

### Participants

Sixteen health professionals were interviewed, most by telephone (1 in person, 1 email): 4 plastic surgeons (PS) (2 F, 2 M), 4 gynaecologists (Gyn) (3 F, 1 M), 3 nurses (N) (educator, theatre, consultant, all F), 3 cosmetic/aesthetic surgeons (CS) (all M), 1 general practitioner (GP) (F), and 1 pelvic floor physiotherapist (Ph) (F). They had a mean of 21.5 years in practice (range 4–37) and were aged 32–76 years (mean 49.4). There were 7 from Victoria, 7 from New South Wales, and 2 from Queensland. All had trained in Australia (some with additional training in the UK or New Zealand) except 2 who had trained in the UK.

Four beauty therapists (BT) (all F) and a body-piercer (Pi) (M) were also interviewed, 3 by phone, 1 in person, and 1 by email. They were aged 33–44 and had been practising for 10–27 years. All lived in Victoria and had been trained in Australia or New Zealand.

Participants are cited or quoted using a 3-component code: their interview number, their profession (as noted above), and whether they are female (F) or male (M); for example ‘1-N-F’. Interviews took from 25 to 48 min (mean 34); each email interview was 2 pages long.

### Discourses

We identified discourses within four themes: *Diversity and the Normal Vulva*; *Indications for FGCS* (Functional, Psychological, Appearance); *Ethical Perspectives*; and *Reasons Women Seek FGCS* (Pubic Depilation, Media Representation, Pornography, Advertising Regulations, Social Pressure, Genital Unfamiliarity). The first three concern the work of professionals and the fourth concerns their speculations on women.

### Diversity and the normal vulva

All participants stated that the appearance of the normal vulva was diverse. Two health professionals named *The Great Wall of Vagina* [29] as a resource to educate patients about genital diversity (12-PS-F, 17-Ph-F), and one nurse used genital images for the same purpose (1-N-F). Some health professionals described explaining to patients that it is normal to have labia minora protrude past labia majora (e.g., 7-PS-F, 12-PS-F) and said that they would reassure women concerned about their genitals; for example:

*I would try to convince them that there is a range of normality, and that normal anatomy is not perfect. I would try to talk to them about the fact that there are false attitudes out there, and I would try to encourage young women to consider that being normal is fine, no matter how their vulva looks.* (11-Gyn-F)

*There’s a massive variation, as with anything. Like in a population, you have a variation of height, or hair colour, or eye colour: it’s the same with the labia. There’s different sizes, and they’re all normal.* (13-Gyn-F)

The body-piercer said, ‘Everyone’s normal; … I don’t understand why you’d look at your inner labia and go, It’s too big; get rid of it!’ He had been consulted by a woman whose sexual partner had queried the normality of her vulva, and asserted:

*The last thing I would ever do is say to someone, ‘Wow! Your junk’s different!’, you know? Because whoever did that to her that caused her such a stigma. I’d never want to be a part of that.* (18-Pi-M)

Healthcare professionals commented on other practitioners:

*I’ve seen some of these documentaries … and the surgeon saying, ‘Oh, we’re going to remove this excess tissue’, or ‘this unnecessary tissue’, or ‘this abnormal tissue’. Well, it’s not abnormal or unnecessary: it was put there for a reason.* (21-Gyn-F)

However, it was evident that ‘diverse’ was not always equated with ‘normal’:

*Some of the women actually have their labia sticking out of their underwear. … Now, you can call them ‘normal’ as well, but they don’t fit today’s world.* (16-CS-M)

Healthcare professionals’ use of pathologising language, such as ‘excess’, ‘abnormal’, and ‘unnecessary’, indicated that, despite the acknowledged diversity, ‘normal’ tended to be applied to a vulva without visible labia minora or clitoris, indicating a pervasive discourse of vulvar diversity as pathological. The medicalised term ‘hypertrophic’ was frequently reported or adopted. Labia minora were also described as ‘enlarged’, ‘giant’, ‘huge’, and ‘asymmetric’, with ‘massive localised gigantism’ or ‘gross asymmetry’, despite the normal asymmetry of all parts of the human body. For example:

*Most people have it [FGCS] because they’ve always had a problem with excessive tissue. You do get a couple of people who’ve had genital problems, for example asymmetry, or where one side is mal-developed or poorly-developed.* (8-PS-M)

Although all non-health professionals denied there was a ‘normal’ vulvar appearance, they were aware of a genital standard or ideal. For example, one beauty therapist reported that women most likely to ask her about whether they were normal had ‘Really, really big labia’ (20-BT-F). The non-health professionals recognised a preference for symmetrical labia minora (18-Pi-M, 19-BT-F) and small labia minora and clitoris (18-Pi-M, 19-BT-F, 20-BT-F). Protruding labia minora were described as ‘an extra set of flaps’ (20-BT-F) and a ‘protruding’ ‘skin tag’ (19-BT-F). The piercer, who was adamant that ‘There’s definitely no norm there’, said:

*As much as I hate saying this, there is kind of a normal presentation. … Not too much outer labia, not too much inner labia, everything reasonably symmetrical, a pronounced but not overly big clitoral hood, that sort of thing.* (18-Pi-M)

### Indications for FGCS

Healthcare professionals without exception said that FGCS should be performed only when there was a clinical indication. However, there was no consensus on what constituted a clinical indication. The discourses of Functional, Psychological, and Aesthetic indications were usually assessed by participants as interconnected.

A complicating factor presented by some surgeons was the difficulty of persuading Medicare that a patient’s genitalia were ‘abnormal’. When applying for a Medicare rebate, doctors were not allowed to send photographic evidence. This, it was argued, forced doctors to describe each woman’s condition, which tended to increase subjectivity in the assessment (10-PS-M, 12-PS-F). It was claimed that the refusal to offer Medicare rebates created an incentive for women to see cosmetic surgeons instead of gynaecologists about their concerns (21-Gyn-F). There were complaints of a double standard in relation to which cosmetic surgeries receive rebates: ‘bat ears’ do, and breast augmentation does with suitable photographic evidence (16-CS-M). The absence of guidelines for what constitutes a clinical indication for FGCS was seen as contributing to ambiguity, leaving assessment ‘open to the interpretation of the practitioner’ (8-PS-M).

### Functional indications

Physical discomfort (such as during exercise or sexual activities) and poor hygiene were nominated as functional problems justifying surgery. One plastic surgeon gave an account that summarised the views of other healthcare professionals:

*From my experience, the clinical indications really are asymmetry, where one side is bigger than the other; pain and discomfort in certain kinds of recreational sporting activities; wearing tight clothing, particularly tight jeans or gym clothes; exercises, particularly sitting on a bike, or cyclists; horse-riders. A lot of patients will complain of those symptoms of pain and discomfort. Pain with sexual intercourse people do have, but it’s not very common. Most people describe more of an awkwardness and inconvenience where the tissues are excessively long, and they get in the way. Obviously, problems, sometimes, with personal hygiene and grooming, can be an issue as well. But I’m sure a lot of people, although there may be a functional component, part of it is also driven by, obviously, the improvement in the aesthetics of it*. (8-PS-M)

Although two beauty therapists claimed that pubic depilation would improve hygiene, only health professionals—two of them— associated FGCS with hygiene. A plastic surgeon stated that women with ‘excessive’ tissue may get ‘toilet paper stuck to the [genital] area’ (8-PS-M), and a cosmetic surgeon outlined the route to infection:

*The urine gets directed back into the vagina, and when the patient stands up having passed urine, allegedly, the urine runs out and keeps the genital cleft moist, which means the next time she has her bowels open, the bugs that live in there have a lovely medium to migrate forward. They colonise the urethra, you get urethra vesical reflux, and as a result you get an incomplete voiding. And all you need is a couple of mls of residual urine in the bladder with bugs sitting there, … and you get recurrent cystitis. And this is far and away the commonest urinary tract infection in little girls and in older women. … I don’t regard doing a labiaplasty on a woman like that as cosmetic surgery, but a great many people out there in the great wide world* would *regard it as cosmetic*. (2-CS-M)

Irritation in tight clothing was given as a ‘significant medical reason’ (15-CS-M) for FGCS; ‘excessive’ (10-PS-M), ‘thick’ (13-Gyn-F), or ‘giant’ (12-PS-F) labia minora were said to cause rubbing, ‘making life uncomfortable’ (12-PS-F).

Discomfort was said to be particularly problematic for women in sport (8-PS-M, 14-Gyn-M, 17-Ph-F, 21-Gyn-F), especially in cycling; FGCS was justified in these circumstances. No-one suggested an alternative to surgery, such as modifying the bicycle seat.

*I would say pain and discomfort are by far and away the over-riding reasons why people are seeking the surgery, and interference with recreational and sporting activities probably count for 75 to 80 per cent of the indications the surgery is performed.* (8-PS-M)

One cosmetic surgeon probed for medical indications when women consulted him about appearance:

*They present saying they’ve got big labia and they want a labiaplasty. … When you sit down and get a detailed medical history out of them, the businesses about irritation in tight clothing, recurrent bouts of cystitis and/or vaginal discharge come to the surface. But they don’t come in complaining of those; they come in saying their labia are too big*. (2-CS-M)

In contrast, a pelvic floor physiotherapist was concerned that FGCS *caused* functional problems and described her response to women who considered surgery:

*We try and talk them out of it, because most of my work is with women who’ve got perineal and pelvic pain, and we just see far too many women who’ve had labial or vulval surgery done for cosmetic reasons who then get scar tissue or pain afterwards.* (17-Ph-F)

Discomfort during sexual activity was cited by a few health professionals as an indication for surgery because ‘large’ labia minora could ‘get in the way’ during sexual activity (7-PS-F, 8-PS-M, 11-Gyn-F), with a possibility of ‘mild bleeding’ after sexual intercourse (13-Gyn-F). (No sexual activity apart from penis-in-vagina was mentioned or implied.) One plastic surgeon reported ‘a patient with labia so large that they tore and bled and she needed to seek medical attention after sex with her partner’ (7-PS-F).

A cosmetic surgeon reported warning women that FGCS is not a panacea for their ‘sex life’:

*It won’t give them a boyfriend. It won’t make the husband feel any better about it all. … Doing the labiaplasty does not guarantee that either one or the other is going to enjoy it more or less.* (16-CS-M)

Other health professionals (such as 12-PS-F) were concerned that FGCS could reduce sexual pleasure or cause pain and discomfort. This plastic surgeon described using histopathology in an attempt to identify cellular reasons for vulvar distress and investigating ‘previous psychiatric or psychological treatment, or sexual abuse in the history’. As the next section illustrates, others linked functional and sexual concerns with psychological matters.

### Psychological indications

There was a clear discourse that poor self-esteem and other psychological problems are indications for FGCS, within which distinctions were blurred between cosmetic and clinically indicated surgery. The poor mental health said to be caused by genital appearance ranged from mild to clinical. Some health professionals claimed that FGCS could improve a woman’s confidence (2-CS-M, 7-PS-F, 21-Gyn-F).

A plastic surgeon compared genitals to breasts, supporting modifications to both areas:

*If they’ve got smaller breasts and they don’t feel comfortable with it, and it’s a self-esteem, self-confidence issue, the same as if their vulval tissue is a bit excessive or hangs down, but it can be symptomatic as well, that it obviously is causing a self-esteem, self-confidence issue.* (10-PS-M)

Another plastic surgeon said that it would be ‘inhumane’ to deny FGCS to a girl with.

*asymmetry of the order of 10 centimetres … when she’s going through her adolescent years, with obviously a lot of self-confidence issues.* (8-PS-M)

It was reported by some participants that women’s self-esteem and confidence had been damaged by disparaging comments from sexual partners. For example, men were said to tell their partners that they prefer ‘a little, cute, small vulva’ (5-GP-F); women

*had basically been told that they had long flaps, as it were. And one in particular had withdrawn, and was particularly isolated socially, wouldn’t go out with anyone, and wouldn’t even consider having a sexual relationship with anybody unless something was done. … On psychological grounds, I performed the labiaplasty in those last few women.* (11-Gyn-F)

However, although it was noted that a woman’s ‘low self-esteem’ could be ‘secondary to a previous male partner giving disparaging comments as to the size of the tissue’ (8-PS-M), it was said to be unusual (8-PS-M, 2-CS-M, 15-CS-M). Nevertheless, even without comment from a partner, it was asserted that ‘a woman’s perception of the appearance of her labia’ (7-PS-F) can disrupt her confidence and her relationships. This plastic surgeon gave as an example:

*A woman who says, ‘I try not to have sex with my husband of 20 years because I’m so embarrassed by the way that my labia looks. If I do ever have sex with him, it’s with the lights out, because I just don’t like the way I look’.* (7-PS-F)

She continued that, although ‘surgery is not necessarily the answer’, it can be ‘a simpler solution’, because:

*Counselling a woman that she just needs to accept that she has very, very large labia, and that the problem is in her head, and her perception rather than her body, is a difficult thing.* (7-PS-F)

At the more severe end of the scale, psychological problems and genital dissatisfaction were sometimes attributed to ‘sexual assault or other trauma’ (4-N-F) or a history of abusive relationships that caused women to feel ‘disfigured and unattractive’ (15-CS-M). Views differed on whether FGCS would solve the psychological problem. Body dysmorphic disorder was also flagged by three health professionals as a potential source of genital distress (8-PS-M, 10-PS-M, 15-CS-M). They emphasised the need to identify unrealistic expectations associated with a psychological disorder before performing surgery.

A gynaecologist expressed concern about outcomes for women seeking surgery for psychological reasons, because:

*The young women that ask are often women that will have issues with vulvodynia or vaginismus, painful sex, anyway. And, you know, I would be concerned that they would still have those ongoing issues, and maybe made worse by having scar tissue in the area.* (21-Gyn-F)

A few health practitioners asserted that a woman’s anxiety and sense of self should be managed before ‘tinkering on the surface’ (5-GP-F), perhaps through referral to a psychologist or psychiatrist (4-N-F, 13-Gyn-F, 15-CS-M). Others, while not referring for psychological help, made their own psychological assessments:

*Every person that comes to us for cosmetic surgery, whether it’s genital cosmetic surgery or what I call cosmetic gynaecology, … you do have to do an assessment of their psychological state, albeit not at the level of a psychiatrist, but at a level of whether they’ve got realistic ideas about their problem.* (10-PS-M)

It could be felt as inappropriate to suggest psychological referral:

*When you’ve come to the doctor, and you are pouring your heart out about intimate issues, and then somebody tells you, ‘Sorry, your problem is psychological; off you go to this psychologist or psychiatrist’, that often doesn’t go down well.* (16-CS-M)

It was said that women rarely took advantage of a referral to a psychologist or psychiatrist (7-PS-F). A cosmetic surgeon reported sometimes ‘giv[ing] in’ to women who might have psychological problems and performing ‘a clitoral recession’ to prevent them from seeking more dangerous solutions, such as ‘clitoral shaft amputations’ (2-CS-M).

### Aesthetic indications

The discourse of aesthetic indications was contested because appearance was implicated in other justifications. For example, ‘hypertrophic’ genital material was identified as an indication for surgery on aesthetic as well as functional and psychological grounds. A plastic surgeon who said that she told women ‘it’s normal to have labia minora that protrude past the labia majora’ also said:

*I will commonly say to them, because it’s true, that I do recognise that their labia minora are quite large compared to the normal variation, and that I can understand why they are wanting to seek surgery.* (7-PS-F)

However, most participants rejected appearance as sufficient justification for surgery. One cosmetic surgeon said:

*If they don’t have any physical complaints—in other words, there’s no chafing, there’s no problem with intercourse, they’re able to do everything that they want to do, except that they don’t like the look of what they have—if I see that their anatomy really is so close to being of no problem at all, then I’m not willing to do anything.* (16-CS-M)

A beautician with a similar opinion was concerned about the implications of FGCS to conform to a limited genital ideal:

*There are women who do it because it’s uncomfortable, and it’s impeding their sexual enjoyment or whatever, but I see that as a whole different thing. We’re talking about labiaplasty to look this homogenous, particular way. … It’s the fact that they’re all the same that concerns me, that there is one, right way to be a good girl.* (19-BT-F)

A GP told of reassuring patients concerned about labial asymmetry by saying:

*The most important thing about your genitals is how much pleasure and joy they give you through sensation; … different-sized labia doesn’t actually reflect that.* (5-GP-F)

The reasons given for ‘improving’ genital appearance by those endorsing it or reporting their colleagues’ views were to make the vulva consistent with a standard, evident in the goals of reducing visible labia minora or clitoris, rectifying asymmetry, and counteracting the effects of aging or childbirth. Participants commented on women who want their genitals to be more ‘pretty’ and ‘perfect-looking’ (4-N-F), or who ‘just don’t like the appearance of their vulvas’ because they think ‘it looks a bit ugly down there’ (2-CS-M). This cosmetic surgeon went on to say that he thought it was ‘a great shame’ for women to feel this way.

Some women were said to be concerned about their vulva postpartum, wanting to ‘put things back to prior to children’ (21-Gyn-F) because they ‘didn’t look like [they] used to’ (17-Ph-F). Beauticians said they understood this desire (19-BT-F, 20-BT-F), with one saying, ‘Why wouldn’t you tidy it up after a baby?‘ (19-BT-F). Another beautician recommended a doctor in Thailand who ‘specialises in reconstruction after birth’ because ‘it’s cheaper’ (20-BT-F). Practitioners were reported as performing surgery to promote a youthful appearance,

*doing fat-grafting around there to make it look plumper and change the shape, and that would probably be a cosmetic procedure, obviously enhancing the vulval tissue to make it look fuller.* (10-PS-M)

This surgeon said he had ‘never done it’ nor had a woman asked him to perform the procedure.

Some participants were puzzled by distress over genital appearance, labelling it, for example, as ‘weird’ (3-N-F) because genitals are ‘not out there’ for people to see (19-BT-F). However, a cosmetic surgeon described dismissing genital anxiety as ‘an insult to women’:

*Just because the whole world doesn’t see them doesn’t mean the woman feels any better about it. I don’t think that that’s fair.* (16-CS-M)

Withholding surgery was seen by some as a violation of women’s autonomy:

*Women should have autonomy over their bodies, and they should have the ability to choose to have plastic surgery or cosmetic surgery for what they genuinely believe that they want to have.* (21-Gyn-F)

*We all have something about us we don’t like, some of us more things than others, but sometimes people really focus on it and it can actually really impede on their life, for whatever reason. If that’s the case, then why not? If you want to do it, you’ve got the money, go for your life!* (6-BT-F)

Women’s autonomy was treated by one cosmetic surgeon in what could be considered a cynical manner, charging inflated costs for FGCS to improve appearance:

*These are young women, usually nulliparous, who will come in saying they just don’t like the appearance of their vulvas, and they want something done about it. … I usually say to them that it’s purely cosmetic, and I load the price up, and still they’re happy to pay for it. … And the reason I do that is to act as a deterrent. … Sometimes I think they go away and find somebody cheaper. … That’s a bit of a worry, but I mean, you know, patients have got to accept a certain amount of responsibility for what they do in these areas.* (2-CS-M)

There were also examples of women’s autonomy being denied or undermined. A theatre nurse reported that male colleagues made disparaging remarks about women under anaesthetic, including that, when ‘the inner labia was below the outer’, surgical staff ‘thought it looked revolting’ (3-N-F). She also reported witnessing ‘several’ instances of surgeons performing labiaplasties without the (anaesthetised) patients’ consent, stating that surgeons were ‘egged on’ by their colleagues ‘to correct’ genitals that they found unattractive (3-N-F).

### Ethical perspectives

Diverse discursive positions were evident on matters with ethical implications. The most extreme was the theatre nurse who described procedures on non-consenting anaesthetised patients. What surgeons justified as ‘helping’ women the nurse perceived as unethical practice based on judgements of women’s bodies.

A gynaecologist described her profession as feeling ‘a little bit split on the topic’, revealing the tension between discourses of women’s agency and the policing of female bodies:

*Women should have autonomy over their bodies, and they should have the ability to choose to have plastic surgery or cosmetic surgery for what they genuinely believe that they want to have. And whether that’s cosmetic surgery on their face or their breasts or liposuction or, you know, if they wanted to have their vagina reshaped to something that they find more appealing. But what we all are concerned about is the reasoning, or the societal reasoning, for why that might be happening.* (21-Gyn-F)

Other ethical matters included doubts about the adequacy of training, unethical advertising, and the absence of appropriate counselling and support for women. For example:

*This is still really a pretty unregulated area of medicine, that people can sort of ‘see one, do one’. And certainly, other specialist colleges have had concerns about the training that these people might have. And again, they promote procedures based on pretty emotive language; you know, ‘sculpting’ and ‘rejuvenating’, and all this sort of stuff, without really paying attention to the risks, the complications, let alone the indications for the procedure. So certainly for me as a practitioner, and certainly as being a member of a college that’s committed to women’s health, we had concerns at a college level about the level of counselling that women had before they had these sorts of procedures; you know, pre-operative evaluations and cooling-off periods and, you know, discussion about other options, and also the sort of hosing down of expectations that patients had about these sorts of procedures.* (14-Gyn-M)

Self-interest and a lack of objectivity were also cited as problematic:

*You just have to be concerned that someone who’s going to make quite a lot of money out of doing these procedures is not going to actually always give you a disinterested answer, or be able to have an objective conversation.* (5-GP-F)

A beautician asserted that surgeons are likely to perform surgery:

*They’re not going to give you a counselling session and some vitamins; they’re going to give you what they know how to do.* (19-BT-F)

One explanation for the failure to sanction medical practitioners for unethical or harmful practice is the secrecy, embarrassment, and perhaps shame surrounding FGCS:

*[Women] don’t really want to discuss it with GPs or anyone else. … They want to try and get right to whoever’s going to fix the problem, with talking to as few people as possible about it.* (10-PS-M)

However, while acknowledging that women may prefer not to discuss the appearance of their genitals with a health professional, a nurse claimed that some women encountered GPs who do not take time to discuss such topics (4-N-F).

A further matter with ethical implications is consideration of risks and benefits of FGCS. For example, two plastic surgeons characterised labiaplasty as a ‘straightforward’ procedure with high patient satisfaction and the risk of complications as ‘pretty unlikely’ (8-PS-M, 10-PS-M). Others described genital surgery as ‘complex’ (12-PS-F), with adverse consequences including bleeding, scarring, and decreased sensitivity during sex (4-N-F, 8-PS-M, 10-PS-M). The physiotherapist said that, although poor outcomes might be ‘not highly common’, she and her colleagues urged women to be cautious:

*It’s common enough for us to then say to patients, ‘Look, when you fiddle with healthy tissue, you do have the potential to give yourself an outcome you might not wish’.* (17-Ph-F)

A plastic surgeon was specific about what she says to women who ask about FGCS:

*It’s not just an amputation of the labia minora. I point out how the anatomy is, and how it looks, and how the pigmentation changes, and how the nerves there—and how it’s an area that becomes engorged, so it’s part of sexual pleasure, so that interfering with all of that, creating neuromas—you know, painful nerve lumps—creating scars can reduce their sexual pleasure. So it’s not something that should be undertaken lightly.* (12-PS-F)

### Reasons women seek FGCS

The discursive construction of women’s reasons for seeking FGCS incorporated the popularity of pubic depilation, media representation, pornography, advertising regulations, social pressure, and women’s inadequate familiarity with the vulva.

The popularity of the Brazilian Wax was frequently cited as a source of genital dissatisfaction: women with a hairless vulva tend to dislike what they see (1-N-F, (5-GP-F), 6-BT-F, 8-PS-M, 10-PS-M, 12-PS-F, 13-Gyn-F, 14-Gyn-M, 16-CS-M, 19-BT-F, 21-Gyn-F). A cosmetic surgeon said that he had not seen ‘a proper pubic patch’ in young women (2-CS-M), and a gynaecologist claimed that, until about 15 years before, women trimmed only ‘the bits that hung out the sides’ (21-Gyn-F). Some participants linked pubic depilation to generational differences, claiming that older women did not have a history of shaving. However, the majority of participants said that women of all ages expressed interest in modifying their genital appearance. Beauticians associated a trend among older women with being able to afford various procedures (19-BT-F, 20-BT-F). A plastic surgeon claimed that older women had ‘put up with’ an unsatisfactory vulva for ‘a long time’:

*And then something, which is often not able to be defined, has had them decide that this year they’re going to fix this problem, because they’re sick of putting up with it, and they want to get it dealt with because it bothers them.* (7-PS-F)

Inaccurate or limited representation in online or traditional media of women’s bodies, especially of the vulva, was cited as another influence on women seeking to modify their genitals (8-PS-M, 10-PS-M, 12-PS-F). Women are given ‘a warped view’ of how the vulva should look (12-PS-F) and come to believe theirs does not look as attractive as those in the media (3-N-F, 4-N-F, 5-GP-F, 8-PS-M).

The media was also said to play a role in publicising the availability of FGCS (7-PS-F, 16-CS-M). Even articles that ‘sensationalised’ FGCS were argued to function as raising awareness (8-PS-M).

*If you don’t know that your problem can be dealt with, then it requires more than an average individual to actually find out that it is being dealt with.* (16-CS-M)

However, a few participants claimed that women were aware of airbrushing and other manipulation of images and therefore not influenced by them (6-BT-F, 15-CS-M).

Pornography did not figure strongly in participants’ explanations for FGCS. There were claims about ‘too much easy access on porn sites, so that’s sort of maybe dictating the way women feel about their genital region’ (13-Gyn-F), and ‘Blokes have got a false understanding of what normal anatomy is, because if they look at porn sites, they think all women should look like that’ (11-Gyn-F). In contrast, there were also statements that, although women might discuss pornography with beauticians, they ‘talk about how fake it is and how airbrushed it is, and la la la la la, but not insofar as comparing genitals of theirs and genitals of those on the screen’ (19-BT-F).

Regulations about representations of women’s genitals in Australian media and advertising were presented as playing a much greater role in influencing women to modify their vulvas. For example, a nurse interpreted legislation restricting depictions of the labia minora and clitoris as implying that variations to women’s genitals are ‘indecent and explicit’ (1-N-F), and a gynaecologist said that the legislation resulted in images that made women’s vulvas look pre-pubertal and was thus inherently ‘paedophilic’ (11-Gyn-F). According to a plastic surgeon, similar legislation is active in the US:

*It’s perfectly acceptable to the censors for people to be committing all sorts of sex acts which most people would regard as unsavoury, but it’s not acceptable to have more than a centimetre of labia minora, and therefore a lot of images that they use are actually doctored, filtered, or actually air-brushed out.* (8-PS-M)

A beautician made a point of telling clients about the ‘outrageous’ legislation restricting images of the vulva, reporting that they are ‘usually shocked’ (19-BT-F).

*They’re like, ‘Oh my goodness. It’s like it’s proof that everything that I have felt bad about all my life about my vagina is not my fault. And it’s not just my imagination; there’s a law about it’.* (19-BT-F)

Social pressure, whether originating in public discourse or personal comments, was also suggested as a reason for women seeking to modify their genitals. Participants reported experiences with women who had been subject to derogatory comments about their genitals from female friends and relatives, a female partner, and male partners; ‘women who had basically been told that they had long flaps’ (11-Gyn-F). Participants also described women who sought surgery while describing their partners as happy with their appearance.

*The word ‘ugly’ comes up a lot more than I would expect. … I ask them, ‘Has somebody else suggested that your genitals are ugly?’. They’ll say, ‘No, no. My boyfriend or my husband tells me that he doesn’t have a problem with them at all.’* (7-PS-F).

There were also claims of more general social pressure:

*While most women will tell me that they’re doing it for themselves, … it’s my perception that … they’re doing it because they want to be more within some part of a beauty ideal that they have obtained somewhere.* (7-PS-F)

The final explanation suggested for the increasing popularity of FGCS was that women were insufficiently familiar with the appearance of the vulva, including their own, and that there is ‘a lack of knowledge of the variations of the shape of the vulva’ (2-CS-M). This lack of knowledge was said to make women vulnerable to idealised and limited aesthetic norms.

*There’s many women who don’t have an acceptance in their head of the idea of looking at their labia with a mirror, which I think speaks to their uncomfortableness with that part of the body, which I don’t think is primarily because they have different labia to other women.* (7-PS-F)

## Discussion

Our aim was to identify discourses that help explain and rationalise female genital cosmetic surgery practices and women’s desire for such practices, and to derive from them possibilities for informing clinical education. We found discursive contradiction around *Diversity and the Normal Vulva*; that *Indications for FGCS* concerned function, psychology, and appearance; that *Ethical Perspectives* were problematic; and that *Women’s Reasons for Seeking FGCS* were given as pubic depilation, media representation, pornography, advertising regulations, social pressure, and genital unfamiliarity.

Although we sought diversity in our participants, we acknowledge a limitation that most were Anglo-Australian and that genital ideals vary with cultural values [30]. Research in other cultural contexts will contribute to more complete and complex evidence of healthcare practitioners’ perspectives on female genital cosmetic surgery. We argue that our results constitute an important component of this evidence.

Despite general acknowledgement of vulvar diversity, a contrary discourse around ‘normal’ or desirable vulvar aesthetics was evident. Although healthcare practitioners tended to describe the genitals of women they have examined in their practices as within the normal range, they frequently used pathologising language (as has been found in advertising [16]) and placed broad caveats on that assessment, in the absence of clear clinical guidance. Positioning within a dominant discourse of vulvar aesthetics, especially in comparisons to cultural norms and stereotypes, seemed to be almost unavoidable.

The three indications for FGCS were almost inseparable. Healthcare practitioners downplayed aesthetics as a primary reason for performing FGCS; nevertheless, the appearance of women’s genitals was considered to be a vital component of their psychological and physical health. Amending the vulva to bring it closer to the minimalist, symmetrical ideal was identified by some as improving sexual confidence and general self-esteem. It is perhaps unsurprising that, within a discursive logic where physical, mental, and aesthetic indications for FGCS are inextricably intertwined, claims of resulting improvement to self-esteem and confidence continue to be made by healthcare practitioners, despite the absence of evidence for such claims [1, 3–5].

Discursive constructions of the ethics of FGCS are complex and, we suggest, entangled with broader media and cultural discourses that position women as both agents and victims of gendered cultural norms in the postfeminist era [31], particularly in relation to what are positioned as women’s ‘choices’ to subscribe or not to still-rigid gendered bodily aesthetic norms [13]. As in the tensions noted in postfeminist media cultures more broadly, participants struggled to make sense of the tension between positioning women as agents who are and should be free to make choices about what they do to their bodies, and concerns over the broader gendered cultural and social discourses that might be influencing and shaping their bodily concerns and decisions. Healthcare practitioners are themselves, of course, subject to the same influences from discourses distributed throughout the cultural milieu. Such discourses include that gender is immutably binary and that women’s bodies must conform to an ideal in which their genitals are invisible. Changes to media laws, in Australia and elsewhere, need to be made to ensure that the prepubertal or Barbie Doll vulva is not the only version available.

We note concerning anecdotes of sexist culture in medicine, in which doctors disparagingly evaluate women’s genital appearance and even perform surgery on the bodies of non-consenting unconscious patients. It should not be necessary to remind surgeons not to perform procedures without a patient’s consent but it appears that this fundamental ethical tenet needs to be emphasised.

Other ethical matters raised by participants include inadequate surgical training, financial incentives engendering conflicts of interest, inappropriate advertising, and inadequate specialist counselling and support for women. We also note diverse views among healthcare practitioners on the risks of genital surgery, with some positioning surgical intervention as a relatively straightforward ‘solution’ to physical and psychological concerns and others naming counter-indications and urging caution to avoid harm.

Given that healthcare practitioners are embedded in a culture that polices women’s bodies so intimately, it is clear that there needs to be continuing education, both undergraduate and postgraduate, to alert practitioners to this matter. Medical practitioners, in particular, contribute to discourses that encourage FGCS by linking genital appearance to mental and physical health [10, 15]. It should not be women’s responsibility alone to resist these discourses. While encouraging women to value genital diversity we need also to counter the widely distributed influences that work against the acceptance of women’s bodies as they are, not as restricted to a limited construct of a non-male body. Plastic surgeons, doctors who practise cosmetic surgery, and male practitioners in particular need to be reminded that it is not their aesthetic judgements that govern surgical practice and that they have no role in policing women’s bodies. Because the construct of binary gender and the associated bodily aesthetics are pervasive, these reminders will need to be sustained and continuing. The systems that shape disciplinary and personal constructs of gender have been well described [32].

Our results reinforce the call from ACOG for clinicians to be able to discuss not only genital diversity but non-surgical treatment options and autonomous decision-making [1]. The concern expressed by participants about the lack of clear clinical guidelines reflects the views of professional bodies internationally [1, 3–5]. More recently, Medicare in Australia has defined ‘labial hypertrophy’ as when ‘the patient’s labium extends more than 8 cm below the vaginal introitus’, without referring to any effect on genital function [33]. This information needs to be contextualised within clear clinical guidelines.

Clinical guidelines should challenge advertising that polices women’s bodies instead of contributing to women’s wellbeing. Guidelines could be informed by consultation with consumers, especially on the use of appropriate language and how to refer for psychosocial support.

It is also important to inform women and the community in general about genital diversity, including by publicising The Labia Library [34]. Vulvar aesthetics are a social construct to which medical practice and opinion contribute and by which they are influenced; education and reform need to occur on all fronts. Resources that not only establish genital diversity but also challenge limited vulvar aesthetics could be developed in consultation with women, healthcare practitioners, mental health specialists, and others with knowledge of social constructs of women’ bodies.

## Data Availability

The dataset analysed for this study is available from the corresponding author on reasonable request.

## References

[1] ACOG Committee on Gynecologic Practice (2020). Elective female genital cosmetic Surgery: ACOG Committee Opinion, Number 795. Obstet Gynecol.

[2] Lowe J, Black KI (2021). Female genital cosmetic Surgery. Aust N Z J Obstet Gynaecol.

[3] Royal College of Obstetricians and Gynaecologists Ethics Committee (2013). Ethical considerations in relation to female genital cosmetic Surgery (FGCS).

[4] Shaw D (2013). Female genital cosmetic Surgery. J Obstet Gynecol Can.

[5] RANZCOG Women’s Health Committee., Vaginal ‘rejuvenation’ and cosmetic vaginal procedures. Aust N Z J Obstet Gynaecol, 2019. 10.1111/ajo.12999 31347147

[6] Australian Government (Department of Health). Commonwealth of Australia. Vulvoplasty (MBS reviews). Canberra; 2014. https://www.health.gov.au/internet/main/publishing.nsf/Content/E393B5FFC5978400CA257EB9001EEC59/$File/Vulvoplasty_Review_Report.pdf.

[7] Royal Australian College of General Practitioners (2015). Female genital cosmetic Surgery: a resource for general practitioners and other health professionals.

[8] McDougall LJ (2013). Towards a clean slit: how medicine and notions of normality are shaping female genital aesthetics. Cult Health Sex.

[9] Schick VR, Rima BN, Calabrese SK (2011). Evulvalution: the portrayal of women’s external genitalia and physique across time and the current Barbie Doll ideals. J Sex Res.

[10] Howarth C (2016). Everything’s neatly tucked away’: young women’s views on desirable vulval anatomy. Cult Health Sex.

[11] Crouch NS (2012). Clinical characteristics of well women seeking labial reduction Surgery: a prospective study. BJOG: An International Journal of Obstetrics & Gynaecology.

[12] Moran C, Lee C (2018). Everyone wants a vagina that looks less like a vagina’: Australian women’s views on dissatisfaction with genital appearance. J Health Psychol.

[13] Elias A, Gill R, Scharff C, Elias A, Gill R, Scharff C (2017). Aesthetic labour: beauty politics in Neoliberalism. Aesthetic labour: rethinking beauty politics in Neoliberalism.

[14] Mowat H (2015). The contribution of online content to the promotion and normalisation of female genital cosmetic Surgery: a systematic review of the literature. BMC Womens Health.

[15] Yurteri-Kaplan LA (2012). Interest in cosmetic vulvar Surgery and perception of vulvar appearance. Am J Obstet Gynecol.

[16] Chibnall K, McDonald K, Kirkman M (2020). Pathologising diversity: medical websites offering female genital cosmetic Surgery in Australia. Cult Health Sexuality.

[17] Moran C, Lee C (2013). Selling genital cosmetic Surgery to healthy women: a multimodal discourse analysis of Australian Surgery websites. Crit Discourse Stud.

[18] Liao L-M, Taghinejadi N, Creighton SM. An analysis of the content and clinical implications of online advertisements for female genital cosmetic Surgery. BMJ Open, 2012. 2(6). 10.1136/bmjopen-2012-001908 PMC3533050 23171607

[19] Zwier S (2014). What motivates her: motivations for considering labial reduction Surgery as recounted on women’s online communities and surgeons’ websites. Sex Med.

[20] Lowenstein L (2014). Physicians’ attitude toward female genital plastic Surgery: a multinational survey. J Sex Med.

[21] Learner HI (2020). Botched labiaplasty’: a content analysis of online advertising for revision labiaplasty. J Obstet Gynaecol.

[22] Reitsma W (2011). No (wo)man is an island—the influence of physicians’ personal predisposition to labia minora appearance on their clinical decision making: a cross-sectional survey. J Sex Med.

[23] Westermann LB (2016). Attitudes regarding labial hypertrophy and labiaplasty: a survey of members of the Society of Gynecologic Surgeons and the North American Society for Pediatric and Adolescent Gynecology. Female Pelvic Medicine & Reconstructive Surgery.

[24] Goodman MP et al. Evaluation of body image and sexual satisfaction in women undergoing female genital plastic/cosmetic Surgery. Aesthetic Surg J, 2016. 10.1093/asj/sjw061 27084062

[25] Harding T (2015). Female genital cosmetic Surgery: investigating the role of the general practitioner. Aus Fam Physician.

[26] Simonis M, Manocha R, Ong JJ. Female genital cosmetic Surgery: a cross-sectional survey exploring knowledge, attitude and practice of general practitioners. BMJ Open, 2016. 6(9). 10.1136/bmjopen-2016-013010 PMC5051499 27678547

[27] Clarke V, Braun V (2017). Thematic analysis. J Posit Psychol.

[28] Potter J, Wetherell M (1987). Discourse and social psychology: beyond attitudes and behaviour.

[29] McCartney J (2011). The Great Wall of Vagina.

[30] Nurka C, Jones B (2013). Labiaplasty, race and the colonial imagination. Australian Feminist Studies.

[31] McRobbie A (2009). The Aftermath of Feminism: gender, Culture and Social Change.

[32] Earp BD (2020). Systems thinking in gender and medicine. J Med Ethics.

[33] www.whv.org.au/resources/labia-library.

[34] www.whv.org.au/resources/labia-library

